# Pretreatment Hematocrit Is Superior to Hemoglobin as a Prognostic Factor for Triple Negative Breast Cancer

**DOI:** 10.1371/journal.pone.0165133

**Published:** 2016-11-16

**Authors:** Bo Chen, Danian Dai, Hailin Tang, Xiaohong Ai, Xi Chen, Xiaoyan Zhang, Zhiyan Li, Xiaoming Xie

**Affiliations:** 1 Department of Breast Oncology, Sun Yat-Sen University Cancer Center, State Key Laboratory of Oncology in South China, Collaborative Innovation Center for Cancer Medicine, Guangzhou, China; 2 Department of Radiotherapy, The First Affiliated Hospital, University of South China, Hengyang, Hunan Province, China; 3 Department of Anatomy, Medical College, University of South China, Hengyang, Hunan Province, China; 4 Department of Gastroenterology, Affiliated Nanhua Hospital of University of South China, Hengyang, Hunan Province, China; University of South Alabama Mitchell Cancer Institute, UNITED STATES

## Abstract

**Background:**

Anemia usually refers to low hemoglobin (Hb) levels. Previous studies indicated that anemia negatively influence the survival in various cancers. Hematocrit (HCT) is the volume percentage of red blood cells in blood, which could indicate anemia in both individuals and populations. This study compared the value of HCT with that of Hb for predicting outcomes of patients who underwent treatment for triple negative breast cancer (TNBC).

**Methods:**

A retrospective study of 293 triple negative breast cancer patients, accepting treatment from January 2004 to December 2009 at Sun Yat-sen University Cancer Center, was conducted. Kaplan-Meier curves and multivariate Cox proportional models were used to calculate disease free survival (DFS) and overall survival (OS).

**Results:**

The cut-off value of HCT was 35.9% determined by X-tile software analysis. The cut-off value of Hb was 12.0 g/dl based on the World Health Organization (WHO) criteria. In univariate analysis, low HCT and low Hb were both significantly associated with decreased DFS and OS. In multivariate analysis, HCT (HR: 0.570; 95% CI: 0.331–0.981, *P* = 0.042 for DFS; HR: 0.456; 95% CI: 0.256–0.813, *P* = 0.008 for OS) was still identified as independent predictor of outcome, but not Hb.

**Conclusion:**

Pretreatment low HCT is independently associated with poor prognosis in TNBC patients. However, HCT was found to be superior to Hb in terms of predicting breast cancer mortality. In the future, large-scale prospective studies or validation studies are needed to verify our findings.

## Introduction

With respect to the leading cause of cancer related deaths in women, breast cancer has drawn increasingly attention[1,2]. Triple-negative breast cancer (TNBC) is a subtype, which lacks estrogen receptors (ER), progesterone receptors (PR) and human epidermal growth factor receptor-2 (HER-2) expression. Compared with other breast cancer subtypes, TNBC is associated with a significantly higher probability of recurrence and poorer overall survival [3,4]. In order to make advances in treatment of TNBC, numerous studies focus on the heterogeneous landscape of individual tumors, developing predictive and prognostic biomarkers that can be accessible/acceptable in clinical.

In past decades, there is a growing body of evidence to support the fact that anemia is a syndrome prevalent in patients with cancer[5]. Nearly 30%–90% patients with cancer have anemia[6].According to earlier reports, anemia is closely related to a worse prognosis in cancer patients and some red blood cell parameters could be used as prognostic parameters in different patients with malignant tumors. For example, Metzger et al.[7] found that a low hemoglobin (Hb) concentration predicted poor survival of pediatric patients with Hodgkin’s Lymphoma. In addition, baseline Hb levels correlate with outcomes of radiotherapy in patients suffering cancer, such as cervix[8], prostate[9], head and neck[10]. Red cell distribution width (RDW), which is a quantitative measurement of the variation of circulating red blood cell size, might be served as a convenient marker to predict the mortality risk of patients with lung cancer[11], endometrial cancer[12] and esophageal cancer[13].Recently, a research indicates that anemia pretreatment in breast cancer patients might deteriorate the pathological response to neoadjuvant chemotherapy as well as survival status[14]. Moreover, in breast cancer, anemic patients (Hb< 12.0 g/dl) had a worse local relapse-free survival (LRFS), relapse-free survival (RFS), and overall survival (OS) than those without anemia (Hb≥12.0 g/dl), even exclude the influence of clinical stage[15].

Hematocrit (HCT)is the volume percentage of red blood cells in blood. It is a part of a person's complete blood count results, along with hemoglobin concentration[16]. As an important red blood cell parameter, HCT could indicate anemia in individuals and populations [17]. Additionally, a small-scale study demonstrated that low HCT is relevant to a poor survival in a group of surgically treated renal cell carcinoma patients[18]. Bissinger et al.[19] found that hematocrit were significantly lower in lung cancer patients compared with healthy controls. A prospective data included 150,912 postmenopausal women was showed that a lower hematocrit level was independently associated with an increased risk of colon cancer at the P < 0.001 level[20]. A study by van Rensburg et al indicated that groups with highest risk for esophageal cancer had mildly depressed hematocrit values[21]. However, no report has connected the alteration of HCT with TNBC. Whether pretreatment HCT has an impact on prognosis in breast cancer patients including TNBC patients is still unknown. Therefore, we performed a retrospective cohort study on TNBC patients with surgical treatment and evaluated the prognosis predicting value of HCT compared with Hb.

## Patients and Methods

### Patient selection

Female patients who diagnosed with triple-negative breast cancer in Sun Yat-sen University Cancer Center between January 1, 2004 and December 31, 2009 were retrospectively studied. Inclusion criteria were as follows: female patients diagnosed with triple-negative breast cancer, with data on routine blood tests including hematocrit and hemoglobin, before initiating any chemotherapy or radiotherapy. TNBC was defined as breast cancer with negative expression of ER (-), PR (-), and HER-2 (-) by immunohistochemistry (IHC). We accepted tumors as being negative for expression of ER or PR with less than 1% of the cells expressing positive ER or PR. HER-2-negativity included tumors with immunohistochemical scores of 0/1+. In this study, a total of 502 records were scanned and 209 patients of those were excluded due to the presence at least one of the following criteria: 1) without follow-up; 2) with coexisting or previous cancers other than breast cancer; 3) received neoadjuvant chemotherapy or radiotherapy before collection of the blood count data; 4) with active infections; 5) with kidney disease or autoimmune disorders; 6) not enough data. All patients were followed up to November 27, 2015 or until death from any reason. The Institute Research Ethics Committee of Sun Yat-sen University Cancer Center approved this retrospective study, and written informed consent was obtained from each participant prior to surgery. All patients were anonymous to analysis. The methods in this study were in accordance with the guidelines of national guidelines.

### Data Collection

Data were collected from patients’ medical charts and pathological reports. As part of the physical examinations, peripheral blood was collected before treatment. HCT and Hb were counted using Sysmex XE-5000™ Automated Hematology System (Shanghai, China).According to the American Joint Committee on Cancer (AJCC) 7th Edition pathological staging was classified [22]. Through outpatient medical records or telephone, the follow-up of patients performed by Department of Follow-Up and Medical Record Management.

### Statistical analysis

All statistical analyses were performed using SPSS 20.0 (SPSS, Inc., Chicago, IL) in the present study. Pretreatment HCT and Hb are expressed as means (± standard deviation), and categorical data were described using numbers and percentages. X-tile 3.6.1 software[23] (Yale University, New Haven, CT, USA) was performed to select the most appropriate cutoff points for the HCT level. The cutoff value for Hb based on the World Health Organization (WHO) criteria. The Chi-squared test or Fisher’s exact test were performed to investigate the associations of HCT or Hb with clinicopathologic variables. The primary end point of the study was overall survival (OS), which was calculated from the time of pathological diagnosis to the date of death from any causes or last follow-up. The secondary endpoint of the study was disease-free survival (DFS), which was calculated from the date of initial treatment to the date of the tumor recurrence or distant metastasis. Survival curves were generated using Kaplan–Meier estimates, and differences between the curves were analyzed by a log- rank test. The univariate and multivariate analyses (Cox proportional hazards regression model) were performed to identify the independent factors relevant to patients’ survival. Multivariate analysis was performed the variables that were found to be significant in univariate analysis. The corresponding 95% confidence intervals (CIs) and Hazard ratios (HRs) estimated from the Cox analysis were regarded as relative risks. For all statistical analyses, *P*< 0.05 was considered statistically significant.

## Results

### Patient characteristics

**Fig 1:**
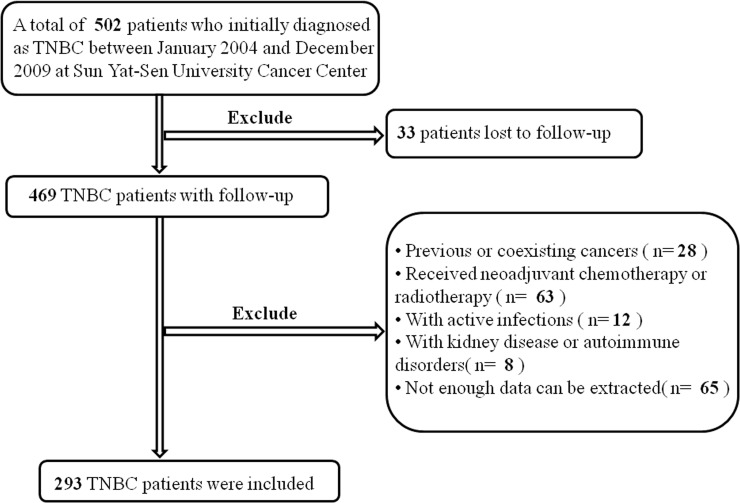
The flow chart of patient selection.

A total of 293 female patients with histopathologically confirmed TNBC were enrolled in this study (Fig 1). The median age for the patients was 47 (range: 22–79) years old. In this study, there was a great diversity of treatment modalities because patients were at diverse TNM stages and with a long diagnosis time span. In enrolled patients,16 (5.5%) patients received radical mastectomy,255(87.0%) received modified radical mastectomy and 22 (7.5%) patients received breast-conserving surgery. After the surgery, a total of 271 patients (92.5%) received adjuvant chemotherapy and 61 patients (20.8%) received adjuvant radiotherapy. Paclitexal-based combination chemotherapy, EC (epirubicin/cyclophosphamide), CMF (cyclophosphamide/methotrexate/fluorouracil), FEC (uorouracil/epirubicin/cyclophosphamide) and CAF/FAC (cyclophosphamide/doxorubicin/fluorouracil) were the mainly chemotherapy regimens. Their median follow-up time was 85 months (range, 2–143 months [censored]), and death occurred in 125 (42.7%) of the 293 female TNBC patients. Other details of features are shown in Table 1. In this study, the mean HCT and Hb were 37.03±3.76%and 12.37±1.30g/dl, respectively.

**Table 1:** The association between HCT, Hb and clinicopathological characteristics.

Variables	Cases (n = 293)	HCT (%)		P value	Hb (g/dl)		P value
		Low No. (%)	High No. (%)		Low No. (%)	High No. (%)	
**Age (years)**				0.839			0.589
≤50	195	72(36.9%)	123(63.1%)		78(40.0%)	117(60.0%)	
>50	98	35(35.7%)	63(64.3%)		54(55.1%)	43(43.9%)	
**Menopause**				0.132			0.149
No	175	70(40.0%)	105(60.0%)		74(42.3%)	101(57.7%)	
Yes	118	37(31.4%)	81(68.6%)		40(33.9%)	78(66.1%)	
**BMI**				0.901			0.728
≤25	223	81(36.3%)	142(63.7%)		88(39.5%)	135(60.5%)	
>25	70	26(37.1%)	44(62.9%)		26(37.1%)	44(62.9%)	
**Tumor Size**				0.876			0.643
≤2.0cm	92	33(35.9%)	59(64.1%)		34(37.0%)	58(63.0%)	
>2.0cm	201	74(36.8%)	127(63.2%)		80(39.8%)	121(60.2%)	
**Tumor status (T)**				0.379			0.768
T1	81	29(35.8%)	52(64.2%)		29(35.8%)	52(64.2%)	
T2	170	58(34.1%)	112(65.9%)		66(38.8%)	104(61.2%)	
T3	21	9(42.9%)	12(57.1%)		9(42.9%)	12(57.1%)	
T4	21	11(52.4%)	10(47.6%)		10(47.6%)	11(52.4%)	
**TNM Staging**				0.004a*			0.162^a^
I	57	19(33.3%)	38(66.7%)		18(31.6%)	39(68.4%)	
II	154	45(29.2%)	109(70.8%)		56(36.4%)	98(63.6%)	
III	72	37(51.4%)	35(48.6%)		35(48.6%)	37(51.4%)	
IV	10	6(60.0%)	4(40.0%)		5(50.0%)	5(50.0%)	
**LN Infiltrated**				0.009*			0.045*
No	150	44(29.3%)	106(70.7%)		50(33.3%)	100(66.7%)	
Yes	143	63(44.1%)	80(55.9%)		64(44.8%)	79(55.2%)	
**Histological grade**				0.957^a^			0.734^a^
G1	3	1(33.3%)	2(66.7%)		1(33.3%)	2(66.7%)	
G2	142	51(35.9%)	91(64.1%)		57(40.1%)	85(59.9%)	
G3	148	55(37.2%)	93(62.8%)		52(35.1%)	96(64.9%)	

Abbreviation: HCT hematocrit, Hb hemoglobin, BMI body mass index, LN lymph node.

a. Using Fisher’s exact test

* P < 0.05, statistically significant.

### Cutoff value and the association between HCT, Hb and clinicopathological characteristics

**Fig 2:**
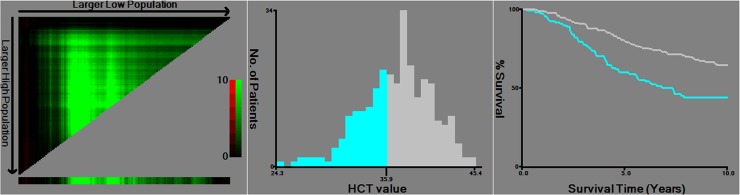
X-tile analysis of prognosis based on HCT. The optimal cut-point in the left panels is shown on a histogram of the entire cohort (middle panels), and a Kaplan-Meier plot (right panels).

Cut-off level for anemia was defined as Hb<12.0 g/dl in females based on the current WHO guidelines. According to previous studies, almost all of them use 12.0 g/dl as the cut-off value for Hb to analyze the impact of pre-operative Hb levels on DFS and OS. Therefore, we choose 12.0 g/dl as the cut-off value for Hb in this study. The optimal cut-point of HCT was 35.9% which was determined by X-tile program (Fig 2). The χ^2^ log-rank value of HCT was 15.91. Enrolled patients were stratified into low- and high- groups according to cut-off points. 107 (36.5%) patients were categorized as low-HCT group (HCT ≤35.9%) and 186(63.5%) patients were categorized as high-HCT group (HCT >35.9%). Similarly, 114 (38.9%) patients were categorized as low-Hb group (Hb<12.0 g/dl) while the remaining 179 (61.1%) patients as high-Hb group (Hb≥12.0 g/dl). Clinicopathological features according to HCT groups and Hb groups are summarized in Table 1. A statistically significant association between low-HCT groups and high-HCT groups in TNM staging (*P* = 0.004) and lymph node (LN) Infiltrated (*P* = 0.009), whereas no correlation with age, menopausal status, body mass index (BMI), tumor sizes, tumor status and histologic grades (all *P*> 0.05). Pretreatment Hb was significantly associated with LN Infiltrated (*P* = 0.045). However, no significant differences were detected between low-Hb groups and high-Hb groups in age, menopausal status, BMI, tumor sizes, tumor status, TNM staging and histologic grades (all *P*> 0.05).

### Survival analysis and the prognostic value of HCT and Hb in TNBC patients

**Fig 3:**
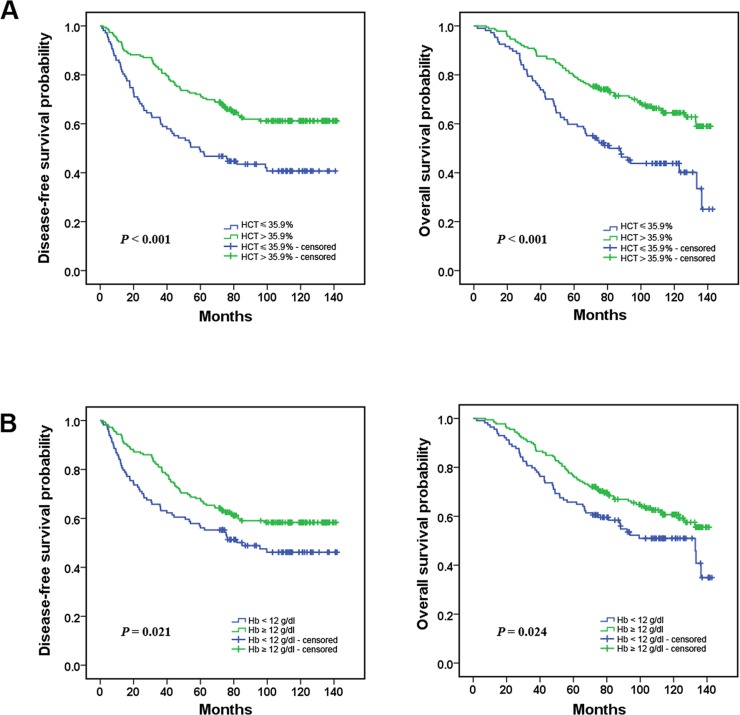
Prognostic value of the pretreatment HCT and Hb levels in triple negative breast cancer patients. (A) TNBC patients with a lower level of pretreatment HCT(≤35.9%) had a worse disease-free survival and overall survival compared with those with a higher level of pretreatment HCT(>35.9%); (B) TNBC patients with a lower level of pretreatment Hb(<12.0g/dl) had a worse disease-free survival and overall survival compared with those with a higher level of pretreatment Hb (≥12.0g/dl).

As the Kaplan–Meier survival curve shown, the low-HCT group patients presented shorter mean months of DFS and OS than the high-HCT group patients (*P*<0.001 for DFS and OS; Fig 3A and Table 2); The lower level of Hb was associated with a lower disease-free survival rate and a lower overall survival rate than the higher level of Hb (*P* = 0.021 for DFS, *P* = 0.024 for OS; Fig 3B and Table 2).

**Table 2:** The DFS and OS of triple negative breast cancer patients according to levels of HCT and Hb.

Variable	Case	DFS (months)			OS (months)		
		Mean ± SE	Median	P-value	Mean ± SE	Median	P-value
Total	293	92.9±3.3	NR		102.7±2.9	136.4	
**HCT**				<0.001*			<0.001*
Low group		75.6 ± 5.6	59.5		86.6 ± 4.9	81.1	
High group		102.6 ± 3.8	NR		111.2 ± 3.3	NR	
**Hb**				0.021*			0.024*
Low group		83.0 ± 5.5	85.0		94.2 ± 4.8	133.0	
High group		98.7 ± 4.0	NR		107.2 ±3.4	NR	

**Abbreviations:** DFS disease free survival, OS overall survival, HCT hematocrit, Hb hemoglobin, NR not reached

* P < 0.05, statistically significant.

For further investigation, univariate and multivariate Cox proportional models were calculated (Table 3). Univariate analysis demonstrated that tumor size (*P* = 0.036), lymph node metastasis (N2 and N3 both *P*<0.001), TNM Staging (Stage III and Stage IV both *P* <0.001), histological grade (G1+G2 versus G3, *P* = 0.003), HCT (*P* <0.001) and Hb (*P* = 0.022) were significantly associated with the DFS in our study cohort. Lymph node metastasis (N2 and N3 both *P* <0.001), TNM Staging (Stage III *P* <0.001; Stage IV *P* = 0.002), histological grade (G1+G2 versus G3, *P* = 0.004), HCT (*P *<0.001) and Hb (*P* = 0.025) were associated with OS. In multivariate analysis, HCT (HR: 0.570; 95% CI: 0.331–0.981, *P* = 0.042 for DFS; HR: 0.456; 95% CI: 0.256–0.813, *P* = 0.008 for OS) was still identified as independent prognostic factor for DFS and OS. However, no statistical significance of the prognostic effect of Hb was observed in the multivariate analysis (*P* = 0.672 for DFS and *P* = 0.226 for OS). Other identified independent prognostic factors for DFS included TNM Staging and histological grade and for OS included lymph node metastasis and histological grade.

**Table 3:** Prognostic value HCT and Hb for disease-free survival and overall survival in triple negative breast cancer patients by Univariate and Multivariate analyses.

Variables	Disease-free Survival				Overall Survival			
	Univariate analysis		Multivariate analysis		Multivariate analysis			
	HR(95%CI)	p value	HR(95%CI)	p value	HR(95%CI)	p value	HR(95%CI)	p value
**Age (years)** (≤50 vs. >50)	1.407(0.992–1.995)	0.056			1.408(0.984–2.015)	0.061		
**Menopause** (no vs. yes)	1.111(0.787–1.570)	0.549			1.114(0.782–1.588)	0.549		
**BMI** (≤25 vs. >25)	1.047(0.706–1.553)	0.817			0.988 (0.655–1.490)	0.955		
**Tumor Size** (≤2.0cm vs. >2.0cm)	1.526(1.029–2.263)	**0.036**	1.020 (0.644–1.616)	0.933	1.501(1.000–2.252)	0.050		
**Lymph node metastasis**								
N0	1 (Reference)		1 (Reference)		1 (Reference)		1 (Reference)	
N1	1.285(0.831–1.989)	0.260	0.973(0.599–1.583)	0.913	1.525(0.974–2.388)	0.065	1.183(0.717–1.950)	0.511
N2	2.916(1.875–4.536)	<0.001*	1.495(0.673–3.320)	0.323	2.979(1.885–4.707)	<0.001*	1.676(0.738–3.808)	0.217
N3	5.237 (2.927–9.372)	<0.001*	2.499 (0.997–6.259)	0.051	7.002(3.861–12.698)	<0.001*	3.567(1.396–9.111)	**0.008**
**TNM Staging**								
I	1 (Reference)		1 (Reference)		1 (Reference)		1 (Reference)	
II	1.612(0.914–2.842)	0.099	1.646(0.833–3.252)	0.151	1.607(0.894–2.891)	0.113	1.503(0.798–2.830)	0.207
III	4.090(2.293–7.295)	<0.001*	2.335(0.865–6.306)	0.094	4.129(2.276–7.490)	<0.001*	1.986(0.789–4.998)	0.145
IV	5.358(2.268–12.657)	<0.001*	2.976(1.054–8.401)	**0.039**	4.130(1.664–10.252)	**0.002**	1.789(0.602–5.314)	0.295
**Histological grade**								
G1+ G2	1 (Reference)		1 (Reference)		1 (Reference)		1 (Reference)	
G3	1.692(1.193–2.401)	**0.003**	1.483(1.042–2.110)	**0.029**	1.701(1.186–2.439)	0.004*	1.497(1.027–2.181)	**0.036**
**HCT** (low vs. high)	0.513(0.364–0.723)	<0.001*	0.570(0.331–0.981)	**0.042**	0.471(0.331–0.670)	<0.001*	0.456(0.256–0.813)	**0.008**
**Hb** (low vs. high)	0.670(0.475–0.944)	**0.022**	1.121(0.662–1.897)	0.672	0.668(0.469–0.951)	**0.025**	1.414(0.807–2.476)	0.226

*Statistically significant prognostic factor identified by Univariate/Multivariate analysis

## Discussion

TNBC is a particular type of breast cancer that is well known to exhibit biological aggressiveness and worse prognosis[24,25]. In present study, we found that low pretreatment HCT is associated with DFS and OS independently in TNBC patients who underwent surgical treatment.

As routine blood investigations are part of first investigation being done prior to surgery for cancer patients, it can help in the assessment of cancer and anemia. Hematologic parameters have been reported to correlate significant with prognosis in patients with advanced malignant disease[26]. HCT is the proportion of blood volume that is occupied by red blood cells. Because the purpose of red blood cells is to transfer oxygen from the lungs to body tissues, HCT can become a point of reference in terms of its capability of delivering oxygen. Moreover, anemia refers to an abnormally low HCT. The HCT test is often used to check for anemia, usually along with a hemoglobin test or as part of a complete blood count[27]. In previous studies, pretreatment anemia, indicated by low Hb levels, was reported to negatively influence cancer patients’ clinical outcome[28,29], including breast cancer[15]. However, little is known about the prognostic value of HCT in TNBC patients.

We retrospectively analyzed 293 consecutive TNBC patients who received surgeries as primary treatments. The cut-off value of HCT was 35.9% determined by X-tile software analysis, a robust graphical tool verified by Yale University, and we believe the cut-off values were reliable and convincing. Similar to previous researches, the cut-off value of Hb was 12.0 g/dl based on WHO criteria. In our study, we found that: Both of low HCT and low Hb were associated with shorter DFS and OS in TNBC. Additionally, in multivariate analysis, HCT was an independent prognostic factor for patients with TNBC, while the prognostic value of Hb could not be confirmed. These results indicated that the pretreatment HCT is superior to pretreatment Hb as a prognostic factor in TNBC patients. One possible mechanism for these results might lie in the fact that anemia might result from the extent of cancer-burden, and may also be caused by comorbidities leading to a decreased survival, such as bleeding, hemolysis or nutritional deficiencies. Interestingly, Erkut et al[30] observed that serum ischemia modified albumin level, a novel biomarker for the detection of cardiac ischemia, was negatively associated with HCT and Hb in patients with acute leukemia. However, the underlying mechanisms have not yet been fully elucidated.

In previous studies, many scholars focused on Hb[31–34], application as an anemia indictor, while few studies explored the relationship between pretreatment HCT and cancer prognosis. As indicated in a previous report by Wang et al.[35],black breast cancer patients compared to white breast cancer patients were found to have significantly lower level of Hb and HCT. Moreover, the black patients seemed to have more aggressive tumors and had a higher proportion of tumor with negative ER and PR status (all *P*<0.001).However, they didn't discuss the prognostic value of HCT. To our knowledge, the present study is the first to analyze the prognostic value of pretreatment HCT in TNBC patients.

Hypoxia, caused by anemia, is one of the possible mechanisms for which HCT may as a prognostic factor of TNBC. Tumor progression is the consequence of complex interactions between the host environment and tumor cells [36]. About 50–60% locally advanced solid tumors may exist hypoxic tissue areas and anemia is associated with a poor tumor oxygenation[29].It has been reported that hypoxia associated with cancer malignant progression and it might select more aggressive tumor clones [29,37]. At a lower HCT level, oxygen transport decreases due to the reduced oxygen carrying capacity, which further contributing to the development of hypoxia[38].In addition, Shamseddine et al [39]reported that a significant drop in mean HCT with a significant decline in insulin-like growth factor-1 (IGF-1) levels was noted at the end of the non-nephrotoxic chemotherapy treatment in breast cancer patients. Meanwhile, IGF-1 signaling could synchronize cell proliferation and maturation during erythropoiesis[40]. Although hypoxia may be a reasonable explanation, we didn't found direct evidence of hypoxia. Thus, this problem needs further investigation.

We must acknowledge that there are several limitations. First of all, this is a retrospective analysis and the data were collected from one medical center that> 99% of our patients were Chinese. Secondly, the enrolled patients underwent surgical treatment by multiple surgeons. Thirdly, we could notexclude other potential causes that affect HCT. Nevertheless, our findings provide a new and valuable clue for estimating condition and evaluating prognosis in TNBC. Since our interesting findings, further studies will be needed to answer following questions: Does HCT have prognostic value in TNBC patients who receive neoadjuvant chemotherapy or radiotherapy? Is anemia caused by chemotherapy associated with poor prognosis in TNBC patients? Could the common treatment options for anemic such as iron therapy and erythropoietic-stimulating agents improve the prognosis of TNBC patients?

## Conclusion

In conclusion, pretreatment low HCT is independently associated with poor prognosis in TNBC patients. When compared to Hb, HCT was found to be a superior marker for predicting triple negative breast cancer mortality. In the future, large-scale prospective studies or validation studies are needed to verify our findings.

## Supporting Information

S1 Table Clinical information for 293 triple negative breast cancer patients. (XLS)
